# Primary Hepatic Perivascular Epithelioid Cell Tumors: From Diagnosis to Treatment

**DOI:** 10.3390/life16091447

**Published:** 2026-08-31

**Authors:** Anna Paspala, Panagiotis Dorovinis, Dimitrios K. Vlachos, Myrto D. Keramida, Dionysios Prevezanos, Konstantinos Kossenas, Nikolaos Machairas, Stylianos Kykalos, Evgenia Kotsifa, Tatiana Driva, Apostolos Angelis, Stratigoula Sakellariou, Georgios C. Sotiropoulos

**Affiliations:** 1 Department of Liver Transplantation & Hepatobiliary Surgery, National and Kapodistrian University of Athens, 11527 Athens, Greece; 2 2nd Department of Propaedeutic Surgery, National and Kapodistrian University of Athens, 11527 Athens, Greecekykalos@gmail.com (S.K.); 3 1st Department of Pathology, National and Kapodistrian University of Athens, 11527 Athens, Greece

**Keywords:** hepatic PEComa, perivascular epithelioid cell tumor, hepatic neoplasm, mTOR pathway, immunohistochemistry, targeted therapy, malignant potential, surveillance

## Abstract

Primary hepatic perivascular epithelioid cell tumors (PEComas) are rare mesenchymal neoplasms that involve both melanocytic and smooth muscle differentiation. Primary hepatic PEComas remain a challenging condition for radiologists, pathologists, and surgeons. Our review provides an updated, clinically oriented synthesis of the available evidence on primary hepatic PEComas, integrating recent developments in diagnosis, imaging, histopathology, molecular biology, prognostic stratification, surgical and systemic treatment, and long-term surveillance. Although the majority of hepatic PEComas generally exhibit a slowly progressive clinical course, a small subgroup of them is characterized by aggressive biological behavior with an increased risk of recurrence, distant metastases, and disease-related mortality. As PEComas can resemble other hypervascular liver lesions such as hepatocellular carcinoma and hemangioma, preoperative diagnosis based on imaging techniques can be very difficult. Furthermore, the final diagnosis is usually made by histopathological examination of the surgical specimen, which reports characteristic expression of both smooth muscle markers and melanocytic markers such as HMB-45 and Melan-A. Although mTOR inhibitors have demonstrated antitumor activity in advanced PEComas, evidence specifically supporting their use in primary hepatic PEComas remains limited. Prognostic stratification, treatment selection, and long-term follow-up of primary hepatic PEComa remain major challenges. Further multicenter studies are needed to improve risk assessment and develop evidence-based guidelines for the management of these rare primary liver neoplasms.

## 1. Introduction

Perivascular epithelioid cell tumors (PEComas) are a rare group of mesenchymal neoplasms characterized by distinctive perivascular epithelioid cells, a cell type initially proposed as the “perivascular epithelioid cell (PEC)” by Bonetti et al. [1]. These tumors exhibit a unique immunophenotype with dual melanocytic and smooth muscle differentiation, representing a defining feature of this tumor family [2]. The PEComa family includes angiomyolipoma (AML), lymphangioleiomyomatosis (LAM), clear cell “sugar” tumors, and PEComa not otherwise specified (PEComa-NOS), as recognized in the World Health Organization classification [3]. PEComas may arise in a wide variety of anatomical locations, including the kidney, lung, uterus, gastrointestinal tract, and liver, reflecting their heterogeneous biological behavior [4]. The liver represents the second most common visceral site after the kidney, although hepatic PEComas remain rare overall [4].

The preoperative diagnosis of hepatic PEComa remains challenging, as these tumors may mimic other hepatic lesions, both benign and malignant, including hepatocellular carcinoma, focal nodular hyperplasia, hepatic adenoma, hemangioma, and metastatic disease [5]. Furthermore, the natural history of hepatic PEComas is not well defined, and their clinical course remains unpredictable due to the limited number of reported cases [3]. Although most PEComas are considered benign, a subset may exhibit malignant behavior, including aggressive growth and metastatic potential [6].

Although several recent reviews have addressed hepatic PEComas, including a pathology-focused review by Amante et al. [7], a recent case-based literature review by Kvietkauskas et al. [8], and a systematic review by Papantoniou et al. [9], important gaps remain in the clinical integration and critical interpretation of the available evidence. The systematic review by Papantoniou et al. provided a comprehensive quantitative synthesis of reported adult hepatic PEComa cases, whereas the present review adopts a clinically oriented narrative approach, integrating recent developments in imaging, histopathological and molecular characterization, prognostic stratification, surgical management, systemic therapy, locoregional treatment, and long-term surveillance [9]. In particular, we incorporate emerging hepatic-specific prognostic models, including the recently proposed risk prediction criteria based on clinicopathological features, and discuss their potential advantages and limitations in comparison with the traditionally applied Folpe criteria. We also distinguish evidence derived from primary hepatic PEComas from evidence extrapolated from PEComas arising at other sites, an important consideration when interpreting systemic treatment strategies. Finally, we highlight unresolved clinical questions and future research priorities, including the need for validated prognostic models, standardized surveillance strategies, international collaborative registries, and prospective evaluation of systemic therapies.

For the preparation of this narrative review, the literature was searched by two investigators using PubMed/MEDLINE, Scopus, and Embase. The search focused on publications addressing primary hepatic PEComas and related PEComa-spectrum tumors, with particular emphasis on epidemiology, clinical presentation, imaging, histopathology, molecular characteristics, prognostic factors, surgical and systemic treatment, and follow-up. Relevant original studies, case reports, case series, reviews, and other pertinent publications were considered according to their relevance to the scope of the review. Reference lists of key publications were also examined to identify additional relevant literature. Given the rarity of primary hepatic PEComas and the heterogeneity of the available evidence, the literature was synthesized narratively rather than quantitatively.

## 2. Epidemiology and Classification

The true incidence of PEComas remains largely unknown, as current knowledge is derived primarily from case reports and small case series due to the rarity of these primary tumors [10]. This limitation is even more pronounced for hepatic PEComas, with approximately 300 cases reported in the literature to date [9]. Available data demonstrate a clear female predominance, with most patients presenting in middle age, typically during the fourth to fifth decades of life [8]. PEComas have also been associated with tuberous sclerosis complex (TSC), a genetic disorder involving mutations in the TSC1 and TSC2 genes and dysregulation of the mTOR signaling pathway [10].

From a pathological standpoint, PEComas are classified among mesenchymal liver tumors within the spectrum of hepatic neoplasms [11]. Due to their epithelioid morphology, their differential diagnosis includes hepatocellular carcinoma, metastatic carcinoma, and melanoma [11]. PEComas are further categorized into benign, malignant, and tumors of uncertain malignant potential based on histopathological features [6]. The most widely used system for risk stratification is the criteria proposed by Folpe et al., which include tumor size greater than 5 cm, nuclear atypia, mitotic activity, necrosis, infiltrative growth, and vascular invasion [6]. Tumors demonstrating multiple high-risk features are more likely to exhibit malignant behavior, although no universally accepted criteria for malignancy exist [6].

## 3. Pathogenesis and Molecular Biology

PEComas are mesenchymal neoplasms composed of perivascular epithelioid cells (PECs), which are usually arranged radially around the vascular lumen. Histopathological examination reveals the partial or complete replacement of normal smooth muscle and collagen within the muscular wall of blood vessels by PECs. Cytologically, they present with a clear to lightly eosinophilic cytoplasm and small, round and normochromatic nuclei [6]. Despite their distinctive morphological features, their exact origin remains uncertain, although neural crest cells are a possible precursor [12].

Melanocytic and muscle markers are co-expressed by PEComas. Melanocytic markers include HMB045 (gp100 protein), Melan-A (MART-1), tyrosinase and microphthalmia transcription factor (MITF), whereas myogenic markers include smooth muscle actin (SMA), pan-muscle actin, muscle myosin and calponin. Among these, HMB-45 and Melan-A demonstrate the highest sensitivity for diagnosing PEComas [6].

PEComas are uncommon and predominantly sporadic, although a subset is associated with the Tuberous Sclerosis Complex (TSC) [13]. Alterations in the TSC1 and TSC2 tumor suppressor genes appear to play an important role in their pathogenesis [6]. The TSC1/TSC2 protein complex acts as a key regulator of Rheb-GTP levels and mTORC1 activation [14]. The proposed mechanism of development for these tumors is based on the two-hit model of inactivation of tumor suppressor genes. A mutated TSC1 or TSC2 allele is accompanied by a second mutation (or loss of heterozygosity) in the normal allele of the gene, leading to complete loss of protein expression. As a result of the loss of either of those genes, the activation of mTORC1 signaling causes dysregulation in cellular growth and proliferation, although this is dependent on a variety of signals [15]. Additionally, TFE3 gene rearrangements have been identified in 25% of the reported cases, through an alternative pathogenetic mechanism involving transcriptional dysregulation [16].

## 4. Clinical Presentation

Since most patients do not have any pre-existing underlying liver disease and therefore do not undergo any routine surveillance, these tumors are discovered most often incidentally, during routine physical examination or imaging [17]. Reported symptoms are non-specific and might include diffuse abdominal pain, discomfort, nausea, vomiting, fever [18]. Moreover, PEComas of large size might cause constipation or even ileus [17] and more importantly, as with other large hepatic tumors, these can rupture thus requiring emergency management [19].

Female predominance has been observed, suggesting a possible hormonal association with PEComas development. Several experimental and clinical studies have described that estrogen may induce tumor growth, as this has occurred during pregnancy in patients diagnosed with PEComas [20,21,22,23]. Although most PEComas exhibit benign characteristics, distant metastases have been rarely reported and may represent the first manifestation of the neoplasm [24]. In the current literature, distant metastases have been described in several sites, including liver, lung, bone, regional and distant lymph nodes, brain, supporting the malignant potential and the aggressive biological profile of a subgroup of PEComas [25,26].

## 5. Imaging Features

**Figure 1:**
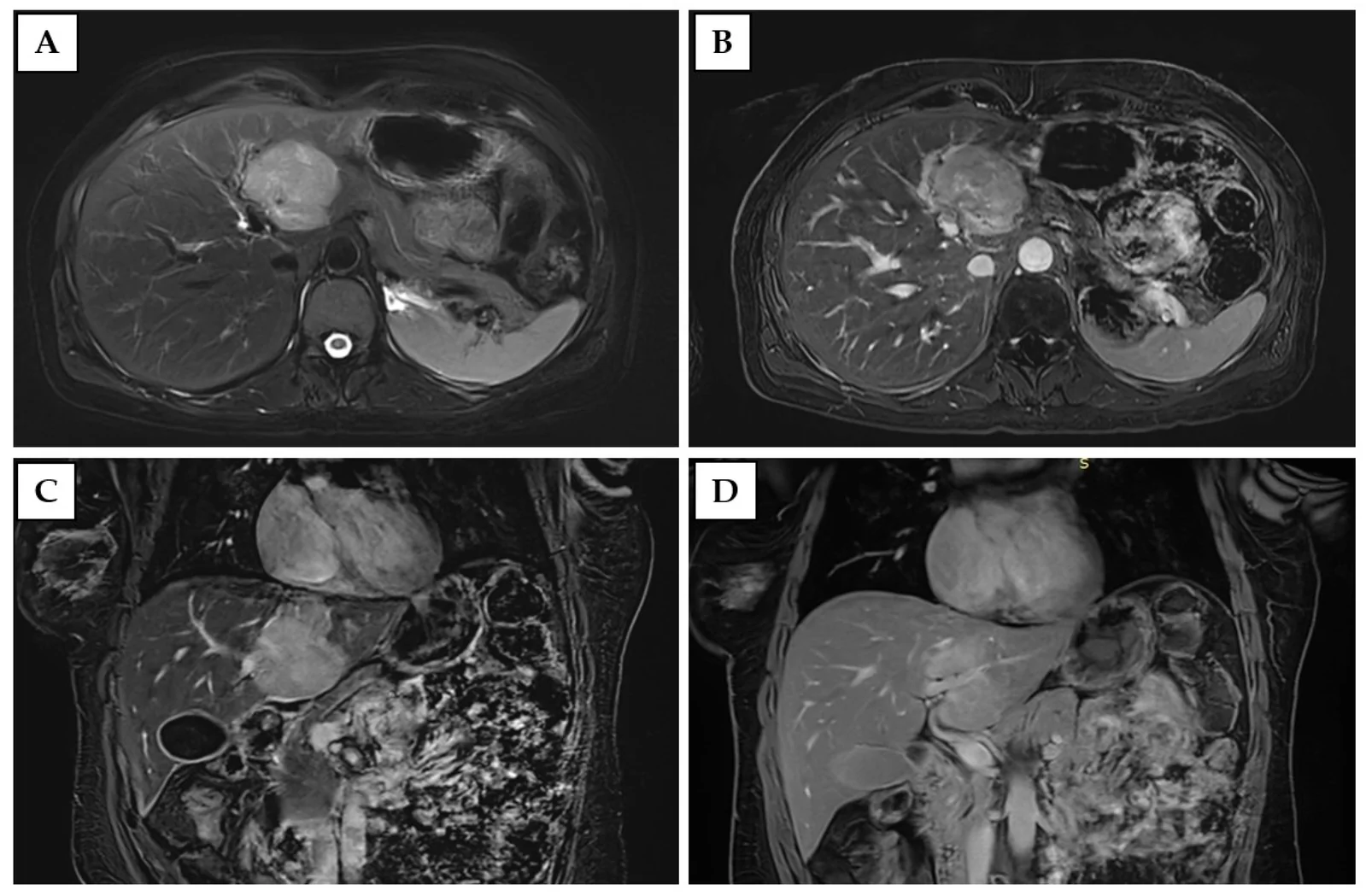
Typical magnetic resonance imaging (MRI) features of primary hepatic PEComa in a 46-year-old patient. Axial T2-weighted (**A**), axial contrast-enhanced T1-weighted (**B**), coronal T2-weighted (**C**), and coronal contrast-enhanced T1-weighted (**D**) images demonstrate a well-circumscribed exophytic hepatic mass arising from the left liver lobe (segments II/III). The lesion is mildly-to-moderately hyperintense on T2-weighted imaging and shows avid heterogeneous arterial-phase enhancement. No definite intralesional hemorrhage, necrosis, or macroscopic fat is evident on the presented images. The mass exerts a mass effect on the adjacent stomach without clear evidence of invasion. Surgical resection was performed, and histopathological examination confirmed the diagnosis of primary hepatic PEComa.

Hepatic PEComas remain difficult to diagnose due to their vague presentation and asymptomatic course. Nonetheless, they lack specific imaging characteristics that would help differentiate them from other hepatic tumors, making a definitive radiologic diagnosis challenging [19]. However, since these tumors have profound arterial vascularization, studies utilizing IV contrast could potentially have increased diagnostic accuracy. Abdominal ultrasound (US) might reveal any type of echogenicity (hyper-, iso-, hypo-echoic) [27] depending on the fat content of the tumor [28]. Doppler imaging can often diagnose central or peripheral hypervascularity [29]. By using contrast-enhanced US (CEUS), primary hepatic PEComas typically exhibit homogenous arterial hyperenhancement with a fast in-slow out (FISO) enhancement pattern [9]. These findings are related to high vascularity of hepatic PEComas. However, CEUS is lacking the ability to determine whether the lesion is benign or malignant [28]. On contrast-enhanced computed tomography (CT), hepatic PEComas typically present as hypodense masses, which similarly to CEUS displays hepatic arterial phase (HAP) enhancement, both within the tumor and to its periphery [19,30,31]. CT can act as an indicator of the underlying pathology but it cannot provide a definite diagnosis regarding the nature of the lesion if not combined with additional clinical or laboratory exams [31]. Magnetic resonance imaging (MRI) plays a role in the initial diagnosis and characterization of these hepatic tumors, through assessment of fat inside the lesions [17]. Hepatic PEComas generally demonstrate low signal intensity on T1-weighted images, mildly elevated signal on T2-weighted images, and increased signal on diffusion-weighted imaging. Moreover, hepatocyte-specific agents reveal marked hypointensity of the lesion during the hepatobiliary phase [19,32]. Although definitive preoperative diagnosis remains challenging, these imaging features combined with hypervascular enhancement patterns might increase radiological suspicion for hepatic PEComas (Figure 1).

In a narrative review Yan et al. stressed the limited accuracy of the above imaging techniques. They attribute an accuracy of 0–33% to ultrasound, 15.7–18.2% to CT and 4.3–22.7% to MRI in order to differentiate PEComas from other liver tumors, as a result of their high heterogeneity [33]. Aiming to increase the diagnostic accuracy of these techniques, the use of a combination has been proposed. Ding et al., in a case series of 79 patients with hepatic AML, reached a diagnostic accuracy of 52%, by combining US, CT, MRI and angiography [34].

Hepatic PEComas are mostly misdiagnosed as HCC, hepatocellular adenoma, focal nodular hyperplasia (FNH), hemangioma or metastatic disease [31]. Several imaging characteristics may help with the differential diagnosis, such as cirrhosis and increased AFP levels in HCC, the classic central scar of FNH, and the significant hyperintensity of hemangiomas [19,31,33]. Lastly the most challenging tumor to differentiate PEComa from is hepatocellular adenoma since they share a lot of their imaging characteristics like the distinct margin, FISO and parenchymal heterogeneity [19].

## 6. Histopathology and Immunohistochemistry

PEComas are rare mesenchymal tumors characterized by the presence of distinctive perivascular epithelioid cells exhibiting both melanocytic and smooth muscle differentiation. Histologically, these neoplasms display a broad morphologic spectrum and may consist predominantly of epithelioid cells, spindle cells, or a combination of both. Tumor cells are typically arranged in nests, trabeculae, fascicles, or solid sheets surrounding a prominent vascular network, often demonstrating a characteristic perivascular growth pattern [6,10,20]. Cytologically, the cells possess abundant clear to finely granular eosinophilic cytoplasm with distinct cell borders and round to oval nuclei containing vesicular chromatin and variably prominent nucleoli. A radial distribution of neoplastic cells around blood vessels is frequently observed and represents one of the most distinctive microscopic features of this tumor family [20].

In hepatic PEComas, the neoplastic cells are commonly polygonal or spheroidal and are organized in nests or sheets separated by a rich sinusoidal vascular network [10]. Additional histological findings may include multinucleated giant cells, extracellular hyaline globules, focal hemorrhage, and varying degrees of cytologic atypia [10,20]. Histopathological features such as increased cellularity, nuclear atypia, elevated mitotic activity, necrosis, infiltrative growth, and vascular invasion may be associated with aggressive behavior; their prognostic significance and limitations in hepatic PEComas are discussed in detail in Section 8 [7,20,35].

**Figure 2:**
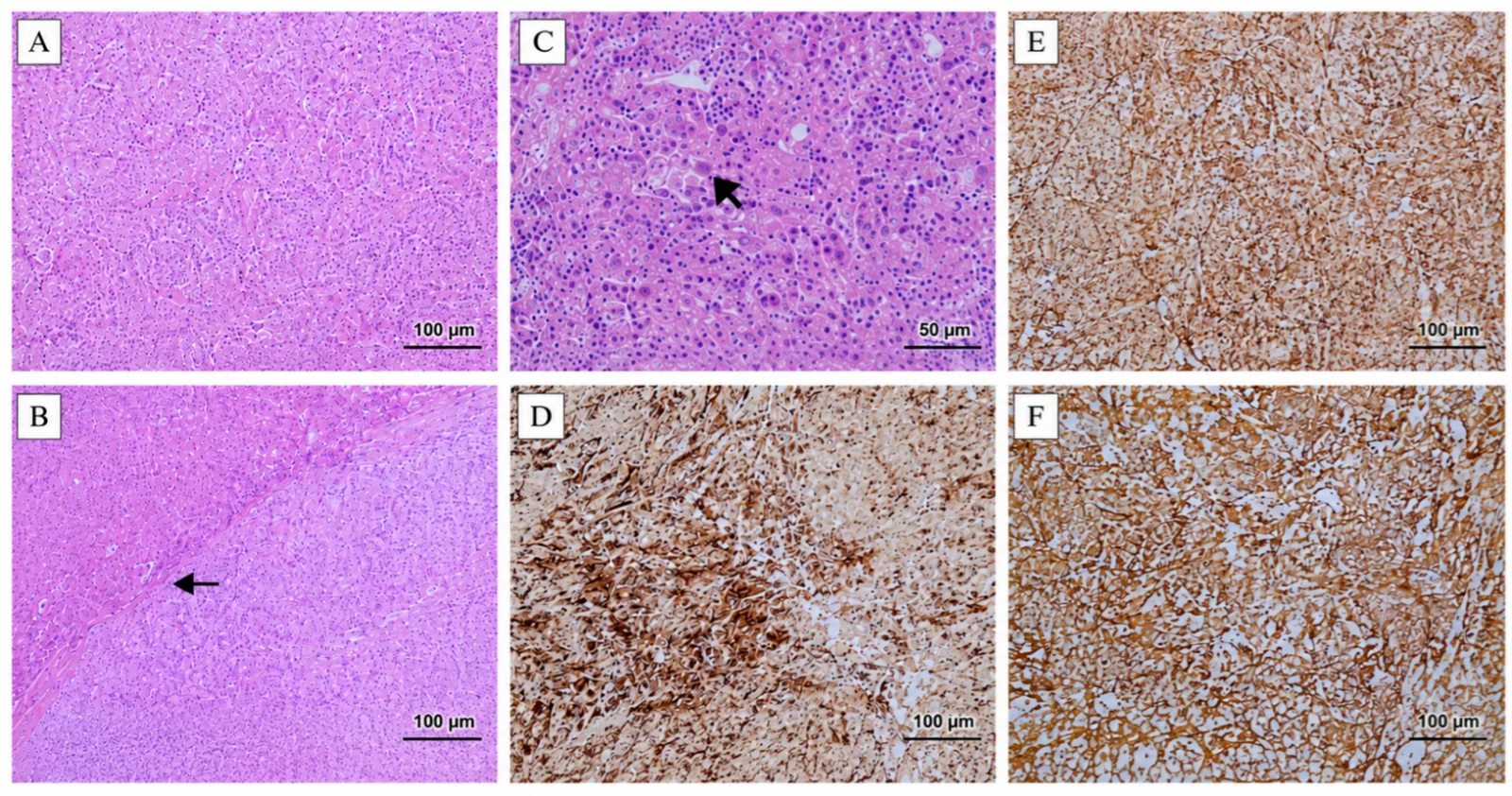
Typical histopathology and immunochemistry features of a resected primary hepatic PEComa in a 48-year-old patient. (**A**) H&E, Low magnification. Tumor composed of sheets of cells, epithelioid with abundant eosinophilic (right) or spindled to epithelioid with clear (left) cytoplasm. (H&E, ×100). (**B**) H&E, Low magnification. At the periphery, the neoplasm is relatively well circumscribed, with focal thin fibrous pseudocapsule (arrow). (H&E, ×100). (**C**) H&E, High magnification. Scattered foci of extramedullary hematopoiesis are admixed with inflammatory cells, with occasional large atypical nuclei (arrow) (H&E, ×200). (**D**) Tumor immunophenotype, MELAN-A. Diffuse moderate to strong positivity of the neoplastic cells for MELAN-A. (MELAN-A immunostaining, ×100). (**E**) Tumor immunophenotype, HMB45. Diffuse strong positivity of the neoplastic cells for HMB45. (HMB45 immunostaining, ×100). (**F**) Tumor immunophenotype, SMA. Diffuse strong positivity of the neoplastic cells for SMA. (SMA immunostaining, ×100).

Immunohistochemistry is central to the diagnosis of PEComa because of its characteristic dual melanocytic and smooth muscle phenotype. Tumors typically co-express melanocytic markers, particularly HMB-45 and Melan-A, together with smooth muscle markers such as smooth muscle actin (SMA), while desmin and other smooth muscle markers show more variable expression [6,10,20]. In hepatic PEComas, diffuse or strong HMB-45 expression together with Melan-A and SMA positivity represents a characteristic immunohistochemical pattern (Figure 2) [10]. Hepatocellular markers such as HepPar-1 are typically negative, which may assist in distinguishing PEComa from hepatocellular carcinoma and other primary hepatic neoplasms [10]. This combined melanocytic and smooth muscle immunophenotype is particularly useful in differentiating PEComa from other mesenchymal and epithelial tumors.

Molecular alterations may provide additional diagnostic and biological information. Alterations involving the TSC1/TSC2 complex, with consequent dysregulation of the mTOR pathway, represent important molecular features of PEComas [6,12,35]. In addition, TFE3-rearranged PEComas constitute a distinct molecular subgroup, typically characterized by epithelioid morphology, strong nuclear TFE3 expression, and reduced expression of conventional smooth muscle markers [16]. TFE3-rearranged tumors may also exhibit a different molecular profile from PEComas associated with TSC1/TSC2 alterations [16]. Although molecular testing is not required for the diagnosis of all hepatic PEComas, identification of TSC1/TSC2 alterations or TFE3 rearrangements may be useful in diagnostically challenging cases and may have therapeutic implications, particularly for the selection of targeted approaches involving the mTOR pathway [10,13,16].

## 7. Diagnostic Approach

**Figure 3:**
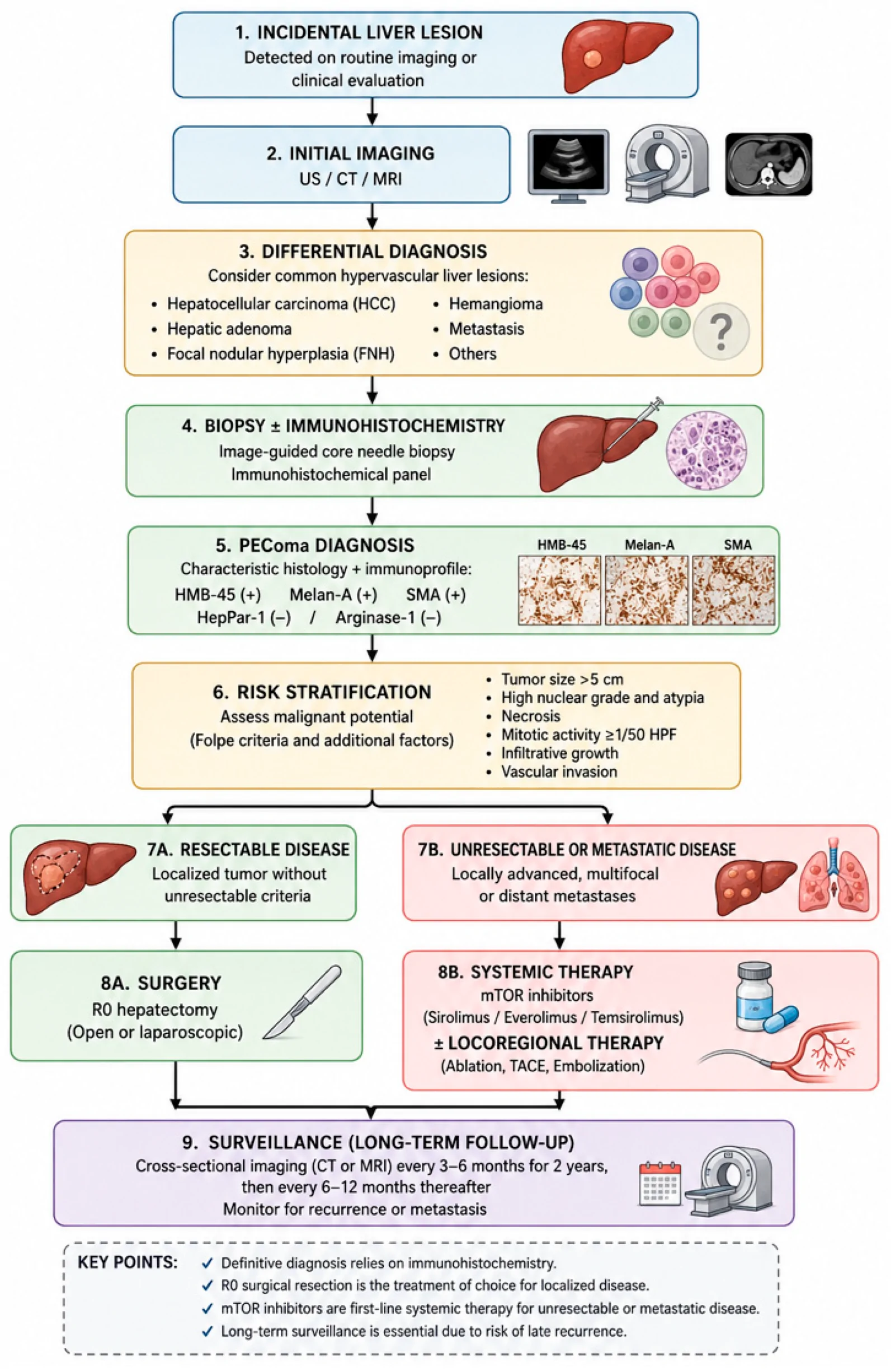
Diagnostic and Therapeutic Algorithm for Primary Hepatic PEComas.

As previously mentioned, preoperative diagnosis of PEComas is extremely difficult and challenging, because there are neither specific radiological features nor pathognomonic laboratory markers to provide a definitive diagnosis [9,31]. The evaluation of alpha-fetoprotein (AFP), carbohydrate antigen 19-9 (Ca 19-9) and carcinoembryonic antigen (CEA), when a liver mass is present, can indirectly aid the diagnosis, since they are commonly negative in PEComas [31]. Moreover, the differentiation between benign and malignant PEComas poses another challenge. Although ^18^F-FDG PET/CT may usually report increased uptake in malignant tumors, [^68^Ga] Ga-FAPI-04 PET/CT would be of profound importance and superiority in detecting PEComas in selected cases [36,37]. Given these limitations, biopsy with immunohistochemical analysis continues to be the most reliable diagnostic method. The pathognomonic expression of both melanocytic markers, such as HMB-45 and Melan-A, and smooth muscle markers significantly improves the accuracy of preoperative diagnosis, when imaging features are inconclusive (Figure 3) [9,20]. Key findings from representative studies addressing prognosis, clinical outcomes, and systemic treatment are summarized in Table 1.

Folpe et al. suggested a prognostic model that remains the most widely used framework for assessing the malignant potential of PEComas [20]. Regarding this model, several histopathological characteristics have been associated with aggressive behavior, including tumor size greater than 5 cm, infiltrative growth pattern, high nuclear grade, hypercellularity, necrosis, vascular invasion, and mitotic activity greater than 1/50 high-power fields. PEComas were classified as benign, of uncertain malignant potential, or malignant according to the presence of these features, with tumors presenting two or more unfavorable characteristics classified as malignant [20]. However, because the original classification was developed predominantly from soft-tissue and gynecologic PEComas, its applicability to primary hepatic PEComas remains debated. Hepatic PEComas may demonstrate discordance between histological appearance and subsequent clinical behavior, with tumors lacking overt malignant features occasionally developing recurrence, while some lesions with worrisome histological characteristics may follow a relatively indolent course [40]. Thus, the Folpe criteria should be regarded as a useful framework for risk assessment rather than a definitive prognostic classification for hepatic PEComas [7,20].

Recent studies have questioned whether the original Folpe criteria are sufficiently accurate for risk stratification of primary hepatic PEComa. Amante et al. emphasized that, although the Folpe criteria were originally developed in a broader PEComa population and have recognized limitations, they remain a useful framework for distinguishing tumors with benign, uncertain, or malignant potential in the absence of universally accepted hepatic-specific criteria [7]. More recently, Yoo et al. analyzed 132 patients with primary hepatic PEComa and related tumors and proposed a liver-specific risk prediction model incorporating tumor size ≥ 7 cm, infiltrative border, mitotic rate > 1/10 mm^2^, necrosis, vascular invasion, and PEComa-NOS histology as worrisome features [39]. Among the 71 surgically resected patients, 4 (6%) were classified as high risk, 31 (44%) as intermediate risk, and 36 (51%) as low risk. Disease progression occurred in one high-risk patient, who developed peritoneal metastases, and one intermediate-risk patient, who developed intrahepatic recurrence, whereas no disease progression was observed in the low-risk group [39]. In a literature review of clinically malignant hepatic PEComa-family tumors, 16 of 26 patients (62%) fulfilled high-risk criteria, while 9 (35%) were classified as intermediate risk [39]. These findings suggest that a composite hepatic-specific model may provide more clinically relevant risk stratification than reliance on individual histopathological features alone. Nevertheless, the model has not yet undergone independent external validation and should therefore be considered promising but preliminary.

Across the available hepatic literature, tumor size, infiltrative growth, necrosis, vascular invasion, and mitotic activity appear to be the most consistently reported features associated with aggressive behavior [6,20,39,43,44]. Tumor size is particularly relevant, with larger lesions more frequently associated with adverse pathological features, recurrence, and metastatic disease. However, no individual parameter has demonstrated sufficient discriminatory ability to reliably predict clinical behavior in isolation. Similarly, cytological atypia or pleomorphism alone should not be interpreted as evidence of malignancy, as these features may also be present in tumors with an otherwise indolent clinical course [9,20,43]. The identification of these overlapping factors across the Folpe framework and the newer hepatic-specific model supports their potential prognostic relevance, but their individual predictive value remains difficult to establish because of the retrospective and case-based nature of the available evidence.

The biological behavior of these tumors appears to be strongly influenced by the histological subtype [20]. Compared to the conventional form of angiomyolipoma, epithelioid angiomyolipoma is thought to represent the variant most frequently associated with aggressive or malignant potential [9,20,39]. It may exhibit infiltrative borders, substantial cytologic atypia, necrosis, and increased cellular proliferation. Nevertheless, focal nuclear pleomorphism or symplastic atypia alone cannot establish malignancy, as tumors with relatively indolent behavior may demonstrate similar histological characteristics [6,7].

The mechanisms driving PEComa carcinogenesis have been better understood thanks to recent advances in molecular pathology [6,10,16]. Dysregulation of the TSC1/TSC2–mTOR pathway plays an important role in PEComa biology and provides a molecular basis for targeted therapeutic approaches [12,13,35]. A distinct molecular subgroup characterized by TFE3 rearrangements has also been identified, typically showing epithelioid morphology and an alveolar growth pattern [10,16]. However, the prognostic significance of TFE3 rearrangement in primary hepatic PEComas remains incompletely defined [10,16].

Given that the vast majority of hepatic PEComas have relatively favorable clinical outcomes, recurrence, metastatic progression, and disease-related mortality remain important clinical concerns. In the systematic review by Papantoniou et al., which included 145 studies and 281 patients with primary hepatic PEComa, the median follow-up was 24 months (IQR, 12–48 months). Recurrence was reported in 17 patients (6%), while eight deaths attributed to PEComa were reported (2.85%); one patient died during the initial hospitalization, whereas 7 patients died as a result of the disease after discharge [9]. However, robust estimates of overall survival or disease-free survival could not be established from the available literature, which is predominantly based on case reports and small retrospective series with heterogeneous follow-up durations and a limited number of outcome events.

Metastatic disease appears to be associated with a less favorable clinical course, but its outcome cannot currently be quantified reliably because metastatic cases are uncommon and are generally reported as individual cases or small series. Reported sites of distant disease include the lung, bone, peritoneum, and, in some cases, the liver [7,9]. However, robust metastatic-specific survival estimates are not available, reflecting the limited number of metastatic cases and the heterogeneity of follow-up in the published literature.

Metastatic or recurrent disease may also occur late after apparently complete surgical resection. For example, Fukuda et al. reported pulmonary recurrence seven years after hepatectomy for a malignant hepatic epithelioid angiomyolipoma [41]. In the hepatic-specific risk prediction study by Yoo et al., disease progression occurred in one high-risk patient who developed peritoneal metastases and in one intermediate-risk patient who developed intrahepatic recurrence, whereas no progression was observed in the low-risk group [39]. These observations support the potential clinical relevance of composite pathological risk features, but the very small number of metastatic and progression events precludes definitive conclusions regarding predictors of metastatic spread or long-term survival. The occurrence of recurrence or metastatic progression, including after prolonged disease-free intervals, supports individualized multidisciplinary management and prolonged postoperative surveillance [9,41].

## 8. Therapeutic Strategies for Primary Liver PEComas

Primary hepatic PEComas are rare tumors characterized by a significant variety of histopathological features, a fact that impacts the choice of the proper therapeutic management [8,9]. As a result, management of hepatic PEComas is based only on case reports, case series, and retrospective studies, which are published in the current literature [9]. The main parameters affecting treatment decisions are tumor size, location of the tumor, malignant potential, resectability, clinical presentation, and the existence of extrahepatic disease [9,44]. Surgical resection with negative margins (R0 resection) is the treatment of choice for these tumors [44]. However, in a subgroup of highly selected low-risk patients, hepatic PEComas might be managed conservatively by following radiological follow-up [8,44]. However, surveillance of probably “indolent” hepatic PEComas remains a challenging decision, as tumor behavior is difficult to accurately be defined, preoperatively [43].

Preoperative diagnosis of PEComas is particularly challenging, since their radiological features can frequently mimic the characteristics of hepatocellular carcinoma, hepatic adenoma, focal nodular hyperplasia, and liver metastases [33,43]. The high percentage of patients who underwent surgical approach for PEComas, can be explained by the fact that conclusive histological diagnosis is mostly made postoperatively [9]. Surgical resection often offers a definitive diagnostic and pathological evaluation in addition to its therapeutic role, both of them are crucial for deciding postoperative follow-up and evaluating malignant potential [9,44].

Radical surgical resection with negative margins (R0 resection) is currently the gold-standard treatment for hepatic PEComas, providing favorable long-term outcomes [8,9]. Kvietkauskas et al. highlighted that R0 resection was performed on nearly 81% of patients, demonstrating the critical role that surgery plays in current management of hepatic PEComas [8]. The extent of hepatectomy might vary from wedge resection to major hepatectomy for either large or near to hilum located lesions. Moreover, the aggressiveness of surgical approach depend on the tumor’s size and location, vascular involvement, and tumor’s malignant potential [9]. Minimally invasive techniques such as laparoscopic liver resection have also shown similar oncological and perioperative outcomes for selected patients [9]. Surgery is usually recommended for symptomatic, enlarging, or diagnostically uncertain lesions in addition to tumors showing radiological or pathological characteristics of malignancy, such as large size, infiltrative borders, necrosis, vascular invasion, rapid growth, and epithelioid morphology [20,44]. Although active surveillance has been proposed for highly selected patients diagnosed with small and asymptomatic PEComas, their unpredictable biological behavior and their potential malignant transformation usually led to surgical resection [8,43,44].

Due to the lack of available data, the role of liver transplantation in primary hepatic PEComa remains highly speculative. In the systematic review by Papantoniou et al., only one patient undergoing liver transplantation for hepatic PEComa was identified [8]. Therefore, there is currently insufficient evidence to determine whether transplantation provides a meaningful oncological benefit in unresectable hepatic PEComa. Concerns regarding tumor recurrence under long-term immunosuppression further limit its potential application. Liver transplantation should consequently not be considered an established treatment strategy and, if contemplated at all, should be restricted to exceptional cases following multidisciplinary assessment and careful exclusion of extrahepatic disease [9,44].

### 8.1. Systemic Therapies

The systemic treatment of primary hepatic PEComa is largely informed by evidence derived from malignant PEComas arising at different anatomical sites, as clinical data specific to hepatic tumors remain extremely limited. This distinction is important when interpreting the available evidence, as the efficacy of systemic therapies demonstrated in mixed-site PEComa populations cannot necessarily be extrapolated to primary hepatic PEComas. Molecular studies have demonstrated that alterations involving the TSC1/TSC2 complex can result in activation of the mTOR pathway, providing a biological rationale for mTOR inhibition in PEComas [12,13,35].

Early clinical evidence with sirolimus demonstrated radiographic responses in all three patients with metastatic PEComa treated in the initial report by Wagner et al. [13]. Subsequently, a multicenter retrospective study of 53 patients with advanced or metastatic PEComa evaluated different systemic therapies and found that mTOR inhibitors were the most active agents, with an objective response rate of 41% and a median progression-free survival of 9 months [33]. In the mTOR inhibitor subgroup, 40 patients were treated, predominantly with sirolimus (32 patients), while 5 received everolimus and 3 received temsirolimus. One patient achieved a complete response, 15 had partial responses, 14 had stable disease, and 9 had progressive disease; 28.2% of patients experienced responses lasting longer than one year [33]. These findings provide clinical support for mTOR inhibition in advanced PEComa, although the retrospective design and inclusion of tumors from different anatomical sites limit their direct applicability to primary hepatic disease.

Data regarding individual mTOR inhibitors remain heterogeneous. Temsirolimus has demonstrated activity in small series and individual patients with malignant PEComa, but the number of treated patients is insufficient to establish a reliable response rate [42]. Similarly, evidence for everolimus in PEComa remains limited and is derived predominantly from small series and individual reports. These data support the activity of mTOR pathway inhibition but do not allow meaningful comparisons between individual agents or the definition of an optimal treatment sequence.

More recently, the prospective phase II AMPECT trial provided the strongest clinical evidence for mTOR inhibition in malignant PEComa. Among 31 efficacy-evaluable patients treated with nab-sirolimus, the objective response rate was 39% (12/31), including one complete and 11 partial responses, while 52% of patients achieved stable disease. The median progression-free survival was 10.6 months, and the median overall survival was 40.8 months. Responses were durable, with the median duration of response not reached at a median follow-up of 2.5 years [38]. Notably, patients with TSC2 mutations demonstrated a higher response rate than those without TSC2 mutations, supporting the biological relevance of the mTOR pathway [38]. However, the AMPECT study included patients with malignant PEComas from different anatomical sites and therefore does not establish equivalent efficacy specifically in primary hepatic PEComa.

Evidence specifically addressing mTOR inhibition in primary hepatic PEComa remains limited. Bergamo et al. reported a case of an initially unresectable hepatic PEComa treated with neoadjuvant sirolimus, resulting in substantial tumor shrinkage after eight months and subsequent successful hepatic resection; the patient subsequently received six additional months of postoperative sirolimus [45]. Although this case illustrates the potential role of mTOR inhibition in selected locally advanced hepatic PEComa, it does not establish a role for routine neoadjuvant or adjuvant treatment. Accordingly, mTOR inhibitors may be considered for selected patients with unresectable, recurrent, or metastatic primary hepatic PEComa within a multidisciplinary setting. However, their efficacy specifically in hepatic disease remains insufficiently established, and there is currently insufficient evidence to define an optimal agent, treatment duration, sequencing strategy, or routine role for neoadjuvant or adjuvant mTOR inhibition [33,38,42,45].

The growing evidence linking molecular alterations to treatment response also highlights the potential role of biomarkers in future risk stratification and therapeutic selection. In the AMPECT trial, TSC2-mutated tumors demonstrated a substantially higher response rate to nab-sirolimus than tumors without TSC2 mutations, supporting TSC2 alteration as a potential predictive biomarker of mTOR inhibition [38]. Beyond TSC1/TSC2 status, emerging molecular studies suggest that additional genomic alterations may contribute to malignant behavior and treatment resistance, although their clinical relevance remains incompletely defined. In particular, TFE3-rearranged PEComas appear to represent a biologically distinct subgroup, while alterations involving TP53, RB1, or ATRX have been associated with malignant behavior in non-hepatic PEComas. Whether these molecular features have comparable prognostic or predictive value in primary hepatic PEComas remains unknown. Prospective multicenter studies incorporating standardized molecular profiling, treatment response, and long-term outcome data will therefore be required to determine clinically meaningful biomarkers and to support more individualized treatment strategies.

### 8.2. Locoregional and Palliative Therapies

Locoregional treatment has occasionally been administered to patients with hepatic PEComas that are metastatic, recurrent, or unresectable. Among these therapeutic modalities, radiofrequency ablation, microwave ablation, transarterial embolization, and transarterial chemoembolization are included [8]. The goal of locoregional therapies is to provide palliative care to patients with metastatic or unresectable disease, while they can be used as a bridging therapy until radical surgical resection [8,9]. Furthermore, transarterial embolization can also be helpful in terms of decreasing intraoperative blood loss during resection of hypervascular or large in size PEComas [44]. However, the available data in the current literature regarding their use remain limited to case reports [8,9].

Although metastatic PEComas are a rare entity, overall survival is unfavorable when distant metastases occur [9]. Management of unresectable, recurrent or metastatic PEComas should be individualized through a multidisciplinary team [8,10]. Recent advancements in molecularly targeted therapies have expanded the therapeutic options for locally advanced hepatic PEComas, whereas surgery is still the standard of care for resectable tumors [10,45].

## 9. Follow-Up

Long-term surveillance is recommended for patients with hepatic PEComas, because, even after R0 resection, their biological behavior is unpredictable. Local recurrence and distant metastases have been reported years after R0 resection, even in lesions without detectable malignant histological features [20,44]. There are currently no defined follow-up protocols, so expert opinion and retrospective studies are the main sources of surveillance strategies. PEComas having unfavorable pathological characteristics, including tumor size > 5 cm, infiltrative growth, epithelioid morphology, vascular invasion, necrosis, significant atypia, increased mitotic activity, and elevated Ki-67 index, are typically advised to be closely monitored [20,43].

Follow-up is primarily based on imaging modalities, and especially contrast-enhanced CT or MRI, whereas MRI is frequently being preferred for detecting small intrahepatic lesions [8]. While annual follow-up during initial years following surgical resection has been recommended, the optimal duration of surveillance is still unclear, as late recurrence might occur many years postoperatively. Patients treated nonoperatively also need frequent imaging, so characteristics indicative of possible malignant behavior, such as rapid enlargement, infiltrative margins, necrosis, or increased vascularity, can be detected [9,43]. Since serum tumor markers are frequently normal in hepatic PEComas, they are normally not useful for follow-up [9]. Further studies are required to improve long-term outcomes and surveillance protocols of primary hepatic PEComas.

## 10. Future Perspectives

Primary hepatic PEComas are increasingly recognized, but many challenges remain regarding diagnosis, prognostic stratification and optimal management. The rarity of these tumors and the preponderance of evidence from case reports and small retrospective series have hampered the development of standardized diagnostic and therapeutic algorithms.

### 10.1. Imaging and Radiomics Advances

A major difficulty in the management of hepatic PEComas is the challenge of a reliable preoperative diagnosis. PEComas often cannot be distinguished from other hypervascular hepatic lesions, such as hepatocellular carcinoma, hepatic adenoma, focal nodular hyperplasia and haemangioma by conventional imaging modalities, including ultrasound, CT and MRI [17,30,32]. Emerging radiomics and artificial intelligence (AI)-based imaging approaches may enhance diagnostic performance through the identification of subtle features that are not easily detected by conventional radiological assessment. Future studies should explore the potential of machine learning algorithms based on multiparametric CT, MRI and contrast-enhanced ultrasound data to facilitate non-invasive diagnosis and improve differentiation from other liver tumors.

### 10.2. Molecular Profiling and Precision Medicine

The increasing knowledge of the molecular biology of PEComa may pave the way for new personalized therapeutic approaches. The most well-known molecular mechanisms of tumorigenesis are changes in the TSC1/TSC2 complex and the consequent activation of the mTOR signaling pathway [10,11,12,40]. However, the molecular heterogeneity of hepatic PEComas is poorly understood. Future genomic and transcriptomic studies may help identify novel biomarkers associated with malignant transformation, recurrence and treatment response. In addition, the prognostic impact of TFE3 rearrangements and other emerging molecular alterations remains to be established [14,34,36]. Comprehensive molecular profiling may eventually allow for the development of individualized treatment strategies and lead to better risk stratification.

### 10.3. Establishment of International Registries and Collaborative Networks

Hepatic PEComas are extremely rare, and single-center experience is insufficient to generate robust evidence regarding long-term outcomes, prognostic factors, and optimal treatment strategies. Established collaborative models in other rare cancers demonstrate the feasibility and potential value of structured multicenter data collection. The European Reference Network on Rare Adult Solid Cancers (EURACAN), for example, has developed collaborative clinical registries aimed at describing the natural history of rare cancers, evaluating prognostic factors and treatment response, assessing treatment effectiveness, and monitoring quality of care [46]. A specific example is the prospective EURACAN registry for epithelioid hemangioendothelioma (EHE), an ultra-rare vascular sarcoma, which was developed as an international collaborative effort to prospectively collect standardized clinical, pathological, molecular, treatment, and longitudinal outcome data [47]. Similarly, the EURACAN observational clinical registry for rare head and neck cancers provides an example of a multicenter cohort designed to prospectively collect standardized clinical and outcome data in a rare-cancer setting [48].

These initiatives provide a potential framework for primary hepatic PEComa, in which an international registry could integrate standardized clinical, radiological, pathological, molecular, treatment, and longitudinal outcome data. Such an approach could facilitate external validation of hepatic-specific prognostic models, identification of clinically relevant biomarkers, assessment of treatment effectiveness, and development of evidence-based management and surveillance strategies. Given the rarity of the disease, international collaboration will likely be essential for generating sufficiently large and well-characterized cohorts to support prospective research and improve clinical decision-making.

### 10.4. Optimization of Therapeutic Strategies and Monitoring

Surgical resection is the mainstay of treatment for localized disease [8,28,33], but many questions remain unanswered regarding the management of hepatic PEComas. The role of active surveillance for small asymptomatic lesions, the best indications for liver transplantation, and the efficacy of minimally invasive surgical approaches still need to be further evaluated. Similarly, the increasing application of mTOR inhibitors [11,36,41,42,44] raises important questions surrounding patient selection, duration of treatment, mechanisms of resistance and the potential role of these agents in neoadjuvant or adjuvant settings.

Finally, there is no current consensus on postoperative surveillance protocols. Prospective studies are required to define the most appropriate imaging modalities, follow-up intervals and duration of monitoring, particularly for patients with tumors with high-risk pathological features. Future developments in molecular diagnostics, AI-assisted imaging and international collaborative research may enable more accurate diagnosis, better prognostic assessment and personalized treatment strategies for patients with primary hepatic PEComas.

## 11. Limitations of the Available Evidence

The interpretation of the available evidence on primary hepatic PEComas is constrained by the rarity of the disease and the consequent predominance of case reports, small case series, and retrospective studies. The available literature is characterized by substantial heterogeneity in patient characteristics, tumor subtypes, diagnostic approaches, treatment strategies, and duration of follow-up, limiting direct comparisons across studies. Publication bias is also likely, as unusual, aggressive, or diagnostically challenging cases may be more likely to be reported than patients with an uncomplicated clinical course. In addition, the evidence supporting prognostic stratification and systemic treatment is frequently extrapolated from PEComas arising at non-hepatic sites, and therefore may not be directly applicable to primary hepatic disease. Although the recently proposed hepatic-specific risk prediction model represents an important step toward improved prognostic assessment, it has not yet undergone independent external validation. Similarly, evidence regarding mTOR inhibitors, liver transplantation, and neoadjuvant or adjuvant treatment remains limited and should be interpreted cautiously. Finally, as the present article is a narrative review, the literature was synthesized qualitatively rather than through a systematic quantitative analysis or meta-analysis. These limitations highlight the need for prospective multicenter studies, standardized reporting, collaborative registries, and external validation of prognostic models to improve the evidence base for the management of primary hepatic PEComas.

## 12. Conclusions

Primary hepatic PEComas remain rare and diagnostically challenging tumors with heterogeneous biological behavior. This narrative review provides an updated, clinically oriented synthesis of the available evidence, integrating recent advances in imaging, histopathological and molecular characterization, prognostic assessment, surgical management, systemic therapy, and surveillance. Particular attention is given to the limitations of applying generic PEComa prognostic criteria to hepatic disease and to the emerging role of hepatic-specific risk stratification and molecularly informed treatment selection.

Nevertheless, the evidence base remains limited by the rarity of the disease and its reliance predominantly on case reports, small retrospective series, and heterogeneous follow-up. Robust long-term survival estimates, validated prognostic biomarkers, and evidence-based treatment or surveillance algorithms remain unavailable. Future progress will require international collaborative registries, standardized clinical and molecular data collection, external validation of hepatic-specific prognostic models, and prospective multicenter evaluation of systemic therapies. Such efforts are essential to improve risk stratification, guide individualized treatment, and establish an optimal evidence-based management strategy for the treatment of patients with primary hepatic PEComa.

## Figures and Tables

**Table 1:** Key evidence from representative studies on primary hepatic PEComas.

Study	Study Design/Population	Main Focus	Key Findings	Main Limitation
Folpe et al., 2005 [20]	Clinicopathological study; 26 PEComas	Prognostic factors	Tumor size > 5 cm, infiltrative growth, necrosis, vascular invasion, and increased mitotic activity were associated with malignant behavior; ≥2 adverse features defined malignant PEComa	Predominantly non-hepatic tumors
Ma et al., 2018 [38]	Retrospective series; 13 hepatic PEComas	Clinicopathological characteristics	Demonstrated substantial histopathological heterogeneity; no recurrence or metastasis was observed among patients with available follow-up	Small cohort and limited follow-up
Yang et al., 2022 [27]	Retrospective single-center study; hepatic PEComas	Diagnosis and treatment	Provided contemporary hepatic-specific data on clinical presentation, diagnosis, treatment, and follow-up	Single-center retrospective study
Papantoniou et al., 2025 [9]	Systematic review; 145 studies, 281 patients	Clinical characteristics and outcomes	Provided the most comprehensive quantitative synthesis of hepatic PEComa; recurrence and disease-related mortality were uncommon but documented	Predominance of case reports/small series and heterogeneous follow-up
Yoo et al., 2025 [39]	Retrospective analysis; 132 hepatic PEComa-family tumors	Prognostic stratification	Proposed a hepatic-specific model based on six worrisome features, with low-, intermediate-, and high-risk groups	No external validation; included PEComa-family tumors
Sanfilippo et al., 2019 [40]	Retrospective study; 53 advanced/metastatic PEComas	Systemic therapy	mTOR inhibitors showed an ORR of 41% and median PFS of 9 months	Mixed anatomical sites
Wagner et al., 2021 [41]	Prospective phase II; 31 efficacy-evaluable patients	Nab-sirolimus	ORR 39%; median PFS 10.6 months and median OS 40.8 months	Mixed-site malignant PEComas
Bergamo et al., 2014 [42]	Case report; locally advanced hepatic PEComa	Neoadjuvant therapy	Neoadjuvant sirolimus enabled subsequent hepatic resection after substantial tumor shrinkage	Single case

## Data Availability

The data underlying this article derive from those included in the relevant published articles.

## Abbreviations

The following abbreviations are used in this manuscript:

PEComas	Perivascular epithelioid cell tumors (PEComas)
AML	Angiomyolipoma
LAM	Lymphangioleiomyomatosis
TSC	Tuberous sclerosis complex
US	Ultrasound
CEUS	Contrast-enhanced US
FISO	Fast in -slow out
CT	Computed tomography
HAP	Hepatic arterial phase
MRI	Magnetic resonance imaging
FNH	Focal nodular hyperplasia
HCC	Hepatocellular carcinoma
SMA	Smooth muscle actin
AFP	Alpha-fetoprotein
TACE	Transarterial chemoembolization
